# Annotation and visualization of endogenous retroviral sequences using the Distributed Annotation System (DAS) and eBioX

**DOI:** 10.1186/1471-2105-10-S6-S18

**Published:** 2009-06-16

**Authors:** Álvaro Martínez Barrio, Erik Lagercrantz, Göran O Sperber, Jonas Blomberg, Erik Bongcam-Rudloff

**Affiliations:** 1 The Linnaeus Centre for Bioinformatics, Uppsala University, Biomedical centre, P.O. Box 598, SE-75124 Uppsala, Sweden; 2 Department of Animal Breeding and Genetics, Swedish University of Agricultural Sciences, Biomedical centre, P.O. Box 597, SE-751 24 Uppsala, Sweden; 3 Department of Neuroscience, Physiology, Uppsala University, Uppsala, Sweden; 4 Section of Virology, Department of Medical Sciences, Uppsala University, Uppsala, Sweden

## Abstract

**Background:**

The Distributed Annotation System (DAS) is a widely used network protocol for sharing biological information. The distributed aspects of the protocol enable the use of various reference and annotation servers for connecting biological sequence data to pertinent annotations in order to depict an integrated view of the data for the final user.

**Results:**

An annotation server has been devised to provide information about the endogenous retroviruses detected and annotated by a specialized *in silico *tool called RetroTector. We describe the procedure to implement the DAS 1.5 protocol commands necessary for constructing the DAS annotation server. We use our server to exemplify those steps. Data distribution is kept separated from visualization which is carried out by eBioX, an easy to use open source program incorporating multiple bioinformatics utilities. Some well characterized endogenous retroviruses are shown in two different DAS clients. A rapid analysis of areas free from retroviral insertions could be facilitated by our annotations.

**Conclusion:**

The DAS protocol has shown to be advantageous in the distribution of endogenous retrovirus data. The distributed nature of the protocol is also found to aid in combining annotation and visualization along a genome in order to enhance the understanding of ERV contribution to its evolution. Reference and annotation servers are conjointly used by eBioX to provide visualization of ERV annotations as well as other data sources. Our DAS data source can be found in the central public DAS service repository, , or at .

## Background

The break-through of bioinformatics provided biologists with overwhelming amounts of publicly available data. To a large extent, biological research has been transformed from being hypothesis driven to data driven in the past few years.

The Distributed Annotation System (DAS) [1] is a simple, lightweight network protocol built on top of the Hypertext Transfer Protocol (HTTP). In some cases, this configuration benefits access to remote servers through firewall protected connections. The client fetches data from the DAS server by sending requests with URLs following a certain command specification. The server responds with a rather simple Extensible Markup Language (XML) message, which follows a strict and well-formed structure. At the time of this writing, the DAS 1.53 complete specification [2] supports a wide range of commands.

Several networks of excellence (NoE) such as BioSapiens, EMBRACE and ENFIN, whose purpose is to create distributed data infrastructures, have recently received funding from the European Union. These collaborating networks are similar in aim but slightly different in biological content. BioSapiens [3] is a large scale and concerted effort to provide genome data annotation from both informatics tools and from experimentalists. ENFIN [4] develops infrastructure, tools and methods to enhance integrative systems biology. The goal of EMBRACE [5] is to integrate the major databases and software tools in bioinformatics using existing web service methods and emerging Grid service technologies.

These NoE encourage data providers to offer data, results or analysis utilizing diverse communication protocols approved by technical committees rather than building big centralized repositories. Discovery and communication with these services, and possibly custom visualizations, should be facilitated to the end user.

DAS is especially suited for:

Providing an easy way to share annotations for a wide range of data types.

Data integration regarding a given reference coordinate system.

Access to the latest version of data while avoiding data mirroring.

The server classification that distinguishes between reference and annotation servers is part of the foundation of the DAS system. DAS relies on a reference object (e.g. a sequence) onto which annotations can be mapped. Every reference object is represented as an entry point named after a stable identifier, such as a chromosome or scaffold name from assemblies, a protein sequence identifier, the name of a gene or a molecular structure identifier. Therefore reference servers can be queried for both the list of entry points they provide and the sequences corresponding to entry points which can be fetched with the *sequence *command. After sequence retrieval, annotations may be collected from a pool of annotation servers which provide data corresponding to the referenced object with the *features *command. DAS clients can collate this information by positional coordinates and display it to the user in an integrated view of the different annotations. With this procedure, as the application logic resides in the client, no coordination among the various information providers is needed. For a better understanding of DAS commands, entry points to reference objects and arguments, see the DAS 1.53 specification [2,6].

The separation between server and client application tiers in the protocol architecture allows a large number of servers supporting various kinds of data and a large variety of clients which collect and display annotations in customized views. Examples of DAS-enabled clients include popular websites like Ensembl [7], Wormbase [8] or GBrowse [9]; applications like IGB [10] or OmniGene [11] and programming libraries like BioJava [12] or BioPerl [13]. On the server side, the number of DAS providers increases: Affymetrix, WormBase, FlyBase, EBI genomic and proteomic, Ensembl, KEGG, Sanger, TIGR, UCSC and UniProt offer data through DAS. As the number of data sources grows, so does the effort required by end users to locate and keep track of information about the contents and locations of various servers. For this reason, a central registry based on the *sources *command of different DAS services was recently established [14]. The DAS registration server [15] implements the protocol and provides information on various DAS sources and their annotated data types, grouping of these sources into coordinate systems, as well as validation and testing of DAS sources. Currently it contains a listing of more than 450 DAS sources.

DAS is heavily used in the genomics bioinformatic community because the original specifications were adapted for working with genomic sequences. An extension (1.53E) [16] to the DAS 1.53 specification was proposed [17] to make the protocol suitable to other types of data. Consequently, coordinate systems were created [16] within this extension. These are normalized reference systems that aid mapping between reference and annotation objects. Their availability provides DAS clients with translation mechanisms into other reference systems and thus capabilities to map information from different data type sources. Its creators thought it to be as a sort of "namespace" [14]. The different parts for defining a coordinate system are *authority*, *type *and *organism*. For a detailed description of these concepts and the possible values to assign, see [18]. Moreover, with the extension commands for server meta information (*sources)*, alignments or mapping between reference objects (*alignment*), and coordinate data of objects such as protein structures (*structure*) were added. Several protein and structure oriented clients such as Dasty2 [19], Spice [20], Proview [21], the CBS DAS Viewer [22] and a DAS compliant JalView [23] were released. Other new commands supporting molecular and protein domain interaction (*interaction*) and volume map data (*volmap*) resulted in new disciplines getting involved into the development of server backends and clients, such as PeppeR [24] for electron microscopy data and DASMI [25] for molecular interaction annotation. Additionally, a protein feature ontology [26] was proposed and adopted by BioSapiens partners.

In spite of the aforementioned developments, there are still genome annotations to unveil. Recent analyses of recently and already sequenced genomes have contributed with annotations such as endogenous retroviruses (ERVs) which are a substantial part of the different genomes. ERVs are estimated to constitute 7% to 8% of the human genome [27] and, together with retrotransposons and solitary LTRs, sometimes over 50% in grain and legume genomes (i.e: wheat, corn, pea) [28,29]. Most scientific sources agree on ERVs being remnants of external infective retroviruses which integrated into the germ-line [30], and are passed on to the offspring in a Mendelian manner. After millions of years since the integration, ERVs could be seen as genome parasites and not as direct players in the biological process of evolution due to highly mutated or removed genes [31]. However, mobile genetic elements (like ERVs) have also conferred greater plasticity to different genomes through transposition, translocation and recombination [32]. In addition they can generate genomic deletions [33], are implicated in causing disease by transposition [34] or xenotransplantation between hosts [35], and can even create new genes or gene families [36] or alter gene expression [37]. For a review to gain insight into the biology of retroviruses and their different functional parts, see [38]. For a review including also a brief background on the different ERV detection tools, see [32].

The study of retroviruses and the importance of their contribution to genomic plasticity may shed light over species development and the establishment and role of endogenous retroviruses in their hosts. With public and open resources such as the proposed DAS annotation server, which is easily integrable along genomic data as reported here, we aim to contribute to the knowledge base of ERVs, enhance retrovirological understanding and facilitate any distributed analysis of human ERVs.

## Results

### Implementation of a DAS annotation server

We have extended a DAS server implementation called ProServer [39], written in Perl [40], with an adaptor module to support retroviral annotation stored in an underlying MySQL relational database. ProServer implements the DAS 1.53E extension and it is actively supported and developed. Source adaptors are plugins into the ProServer interface and produce the response for the various DAS commands. The commands implemented in our server were *features*, in order to respond to a request for annotation data, and *stylesheet*, which gives instructions to guide the client on the visualization of a retrovirus. These commands were programmed in the adaptor class to support the retroviral data being served.

ProServer also facilitates at a higher level the necessary mapping between well formed XML DAS formats and the internal Perl data structures. Source adaptors manage and return data within Perl data types whereas the ProServer stub mechanism converts these structures into the correct XML format.

**Figure 1:**
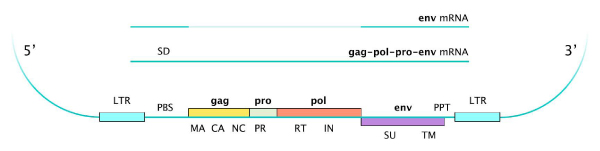
**Provirus structure**. Based on the splice donor-acceptor sites, putative mRNA transcription for the retrovirus with simple replication strategies is shown. Putative viral genes with open reading frames: gag (MA, matrix; CA, capsid; NC, nucleocapsid); pro (PR, protease); pol (RT, reverse transcriptase; IN, integrase); env (SU, surface protein; TM, transmembrane protein). Other parts contained: PBS, primer binding site; SD, splice donor; SA, splice acceptor; PPT, polypurine tract.

**Figure 2:**
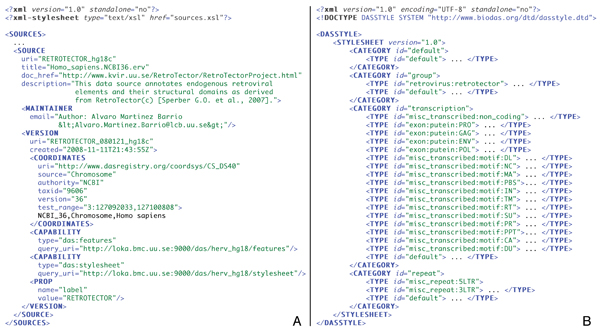
**Left: Example of the DAS sources response in our server**. To understand the configured meta-information of the DAS sources in our server, follow the legend in Figure I of Prlić et al., 2007. Right: Example of a DAS stylesheet response in our server. XML message shown only until TYPE level in order to distinguish better the different categories and types supported. The glyphs are folded into their respective types.

The visualization style was categorized into types according to the different parts that a retrovirus may incorporate (Figure 1). A description of our DAS source, the different categories and types for annotations is given in Figure 2. Every category and type was named according to the latest DAS style specification [41]. Inside each category, glyph forms and colors were chosen following the Document Type Definition (DTD) [42] stylesheet as accurately as it is possible to graphically represent retrovirus parts. Features are annotated at a chromosomal level for the NCBI_36 assembly of the *Homo sapiens *(Figure 2).

The DAS server consults a MySQL relational database containing ERV sequences annotated by virtue of an *in silico *genomic retroviral detector called RetroTector [43]. The source module retrieves the retroviral chains in the genome segment being investigated and their information is computed. Thereafter, the relevant data structure is returned to the server which translates and packages it into an XML response.

**Figure 3:**
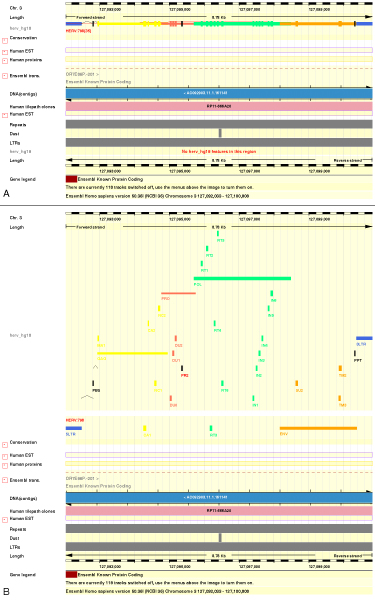
**ERV present in the human genome (chr3:127092033,127100808) as displayed by EnsEMBL contigview in a track labeled herv_hg18 (placed at left part)**. A) After the DAS source was added and configured with features grouped B) Here the DAS source has been configured with ungrouped features and unlimited number of shown features. Color coded features can be appreciated (LTRs – royalblue; gag and motifs associated – yellow; pro and motifs – coral1; pol and motifs – springgreen; env and motifs – orange).

To test the well-formedness of the XML messages and the integration with a standard DAS client, we added our data source to the EnsEMBL genome browser. The process to add a DAS source to EnsEMBL is explained in [44]. For the purpose of visualizing the different parts of a retrovirus, several well characterized ERVs were tested. Specifically the provirus with highest score assigned by RetroTector in the NCBI36 assembly. This HERV is an HML2-like ERV located in chr3:127092033,127100808 (Figure 3).

### Implementation of a DAS visualization client

To best support the capabilities of the DAS *stylesheet *command and bring accuracy to the annotations that our server provides from the different ERV parts, an application was modified to support visualization of DAS 1.53E sources. At the moment of writing, only visualization of genome oriented sources - like ERV annotations - is possible.

Our application, called eBioX (Lagercrantz *et al.*, in preparation), is a user friendly desktop program delivering a suite of open source bioinformatics programs and utilities in a easy to use manner for the non computer literate researcher.

**Figure 4:**
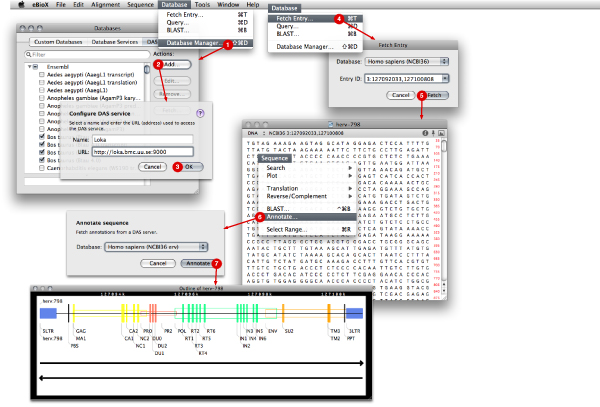
**eBioX integration and visualization**. 1–3) DAS directory service after some sources had been configured 4–5) Fetch entry menu and acceptor window 6) Annotation menu and acceptor window 7) eBioX outlines a view a retrovirus in the annotated region of the human genome (chr3:127092033,127100808).

The application already supported a catalog of database services, where references to external data sources could be added by the user, but this catalog has been extended to support DAS servers and repositories. A DAS repository can be added using any HTTP URL pointing to the service. The added services are automatically queried for information regarding available DAS sources and the associated stylesheet. This information is periodically updated and cached locally for quick access. Users can select a subset of the sources that will be active when retrieving reference sequences or annotations (Figure 4). A few valuable repositories come pre-configured in the default installation.

When the data sources configuration is completed, our application is ready to obtain a reference sequence. For that purpose, the "Fetch entry" command will show a drop down menu to interrogate a list of possible sources (Figure 4). At this stage only reference sources are shown and queried. Parameters for the reference server should adhere to the usual DAS location format: stable_identifier: start_position, end_position [2]. A window containing the retrieved sequence will appear. Annotations can now be added by choosing "Annotate..." from the "Sequence" menu. The program iterates through the sources selected in the database catalog, and finds those matching the coordinate system with the downloaded reference sequence. Only matching annotation sources are shown in a drop down menu (Figure 4) where any individual source can be selected. The corresponding server is then queried for annotations which apply to the sequence, and retrieved annotations will be outlined in a new visualization window in front of the reference sequence. Several annotation sources can be selected in this way to enhance or compare annotations.

With help of this extension in eBioX, we downloaded the genome area corresponding to the same previously annotated retrovirus (chr3:127092033,127100808) from the reference server; an ERV that is likely the most complete ERV in the latest human assembly. Finally, we annotated the sequence using the reported DAS source and displayed it along our reference sequence (Figure 4).

### Applications

**Figure 5:**
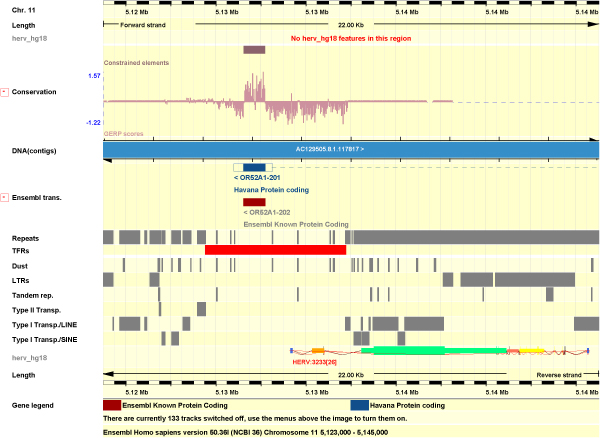
**Human transposon-free region hs11.65 overlapping endogenous retrovirus HERV:3233 in the human genome (chr11:5123000,5145000)**. The horizontal red bar represents hs11.65 (see labels in the left part of the figure). The protein coding gene OR52A1 with two alternative transcript forms is presented in blue and rust colors. The HERV:3233 annotated is displayed in a track called herv_hg18.

Simons et al. state that the human genome contains 856 transposon-free regions (TFRs) with a size over 10 kb and 9203 TFRs over 5 kb [45]. A complete ERV ranges between 7–11 kb long and is flanked on both sides by two long terminal repeats (LTRs) formed during reverse transcription. Our aim was to review if there would be any of the detected ERV among the predicted TFRs that would have been treated as a tandem repeat with all the internal provirus coding regions being considered as a simple TFR. In particular, we had our reservations about the predicted 10 kb TFRs since their size is very similar to typical ERVs. We downloaded a file linked at [46] describing human transposon-free regions (TFRs) in .bed format, parsed the hg18 coordinates reported and combined them into a request as a final test to our server, aiming to determine whether there were ERV annotations overlapping onto their predicted loci. The 10 kb regions were totally transposon free, none of our annotations overlapped. Interestingly, we found 15 ERVs which overlapped over as many 5 kb TFRs regions distributed in 7 different chromosomes (Additional File 1). These ERVs had an average score of 397 assigned by RetroTector. One of them, scoring as high as 722. HERV:3233 is placed in  chromosome 11 and it is a typical HERV-H with gag, pol and env which indeed contains a fairly long open reading frame (ORF). The LTR divergence of the HERV-H element (41%) indicates an old integration in the genome. The human area (chr11:5123000,5145000), including the 6.2 kb TFR identified as hs11.65, is depicted in EnsEMBL (Figure 5) containing our annotation track by the .bed file annotations (Additional file 1).

The performance of our server during the TFR search was measured as the time (in milliseconds) from the moment the request was made until the moment the response arrived. The resulting time was corrected for the network latency and divided among the number of requested TFRs as a measure of the internal server performance (no adjustments were made for the server workload). A search for 856 10 kb regions had an averaged delivery time of 283 ms per request whilst the 9203 5 kb TFRs were interrogated at 406 ms per query.

## Discussion

The data source described here serves our aim to provide a comprehensive array of resources for the study of ERVs, with additional plans to develop a future database supported by a reference server and to offer a broader range of species' annotations. When this database is ready, our past annotations will be updated. The investigation presented here is also directed towards making ERV annotations available, like the public genome databases, and contribute to their visualization along genomes in order to understand the function of mobile elements and mammalian genome evolution. Similar efforts to create an ERV database are HERVd [47], RetroSearch [48] compiling annotated HERV ORFs in the human genome [49], plus the RepeatMasker [50] track at the UCSC genome browser [51], which builds on RepBase [52]. RepBase is a repeat-based annotation system, which annotates repeated sequences by following a nonsystematic nomenclature and containing rather little retroviral information. Retroviral sequences are often fragmented after a RepBase classification so it is hard to see the whole proviral structure. However, RepBase is a useful repository once these limitations are understood.

Our data set contains annotations by RetroTector [43] which fairly accurately resemble the real endogenized proviral structure.

The reported  ERV server builds upon DAS, a lightweight protocol for data exchange and distribution.  It facilitates the integration of our ERV data set with visualization applications and the most common DAS-compliant genome browsers.  The DAS protocol has reached a good level of technological maturity and wide acceptance by the community, especially by bioinformaticians. DAS continues to be extended [16] with the support of different framework projects facilitating the adoption by new research communities. Both electron microscopy [53] and protein interaction [54] groups have committed themselves to develop new extensions recently. Before the 1.53 extension, a group working in the astrophysics field [55] used DAS to exchange annotations of reference entities such as celestial objects.

We extensively support the specification of DAS/1 in the development of our resource. We also appreciate the idea of coordinate systems as introduced with the 1.53E extension because it has the potential to improve integration and standardization of the different DAS sources concerning authority, type and organism names. However, we have foreseen some possible conflict between authorities among the various protein databases and, especially, nucleotide databases containing the same sequence but use different accession numbers, identifiers, assembly names or versions. We realize how important it will be that a client can correctly identify mappings between annotation sources and reference sources without user intervention to automatize the data annotation process. eBioX does this task by taking the coordinate system information into account when fetching annotations though this approach fails for some third party annotation sources with missing or misleading coordinate system information. Furthermore, several important DAS servers still support only the legacy command *dsn *[2,15], which provides no coordinate system information. No effort has been yet made to integrate DAS servers using this deprecated command into eBioX. These obstacles prevented eBioX from using the whole annotation power of the DAS system, but there are potential solutions to this situation. One could build an ontology on top of the coordinate system similar to the one already implemented by BioSapiens regarding the protein feature annotation [26] in order to relate the different reference providers. Moreover, BioSapiens proposed to use MD5 digests instead of an incremental version number to reflect sequence changes [17]. An MD5 digest would be equal for the same sequences in two different authorities regardless of naming practices. Consequently, both the reference object and the corresponding annotations would be related by an identical digest. Unfortunately we have not seen this mechanism implemented in any server at the time of this writing.

The DAS protocol seems to have a promising and sustainable future as a protocol for data exchange. A newly released version called DAS/2 began in July 2004 funded by a 2 year NIH grant [56]. Several data services supporting DAS/2 are already available. Our client should be able to read and understand DAS/2 meta-information about data sources as the extended command *sources *follows the DAS/2 specification. Since November 2006 the retrieval part of the DAS/2 protocol is considered stable but our client is not compatible yet. The DAS/2 protocol itself is not backward compatible with the DAS/1 version and a proxy server under development will facilitate the task of accessing DAS/1 servers by DAS/2 clients. Meanwhile, existing DAS software will continue supporting DAS/1.

Although DAS sustainability and usage seems to be granted, a broad array of technologies specialized for data distribution such as XML Remote Procedure Call (XML-RPC), Representational State Transfer (REST), Simple Object Access Protocol (SOAP) and Extensible Messaging and Presence Protocol (XMPP) [57,58] are alternatives to DAS. DAS is a variant of REST with restrictive well-formedness in its XML responses. Both REST and DAS could be seen as subsets of XML-RPC which is in turn a subset of its successor SOAP.

Protocols that are based on HTTP (e.g. REST, DAS, SOAP and XML-RPC) allow for easy communication through proxies and firewalls compared to previous remote procedure protocols (e.g. CORBA and Java-RPC), which favors integration of resources between organizations. DAS is a good option to replace most of the data stored in flat files and databases because of its easy implementation and availability of many public clients, servers and libraries. Therefore DAS is well adapted for serving pre-calculated data, while SOAP is frequently used to request remote calculation of data and message exchange. The EMBRACE technical recommendation [59] adopted SOAP as model for a communication protocol and message exchange. The data types in SOAP are based on fixed contracts between servers, using Web Service Description Language (WSDL) and XML Schema Definition (XSD), which enables automatic validation of syntactic correctness of the messages. SOAP specifies a strict envelope/header/body message structure which is serialized by a client peer and deserialized at the server side. Because of its verbosity in the XML format, SOAP can result in a performance reduction when transmitting large messages. Typical scenarios of large data messages in bioinformatics comprise, for instance, when assembled  genome sequences or result from analysis tools are sent. In general, the usage of XML syntax in messages has both advantages and drawbacks. Whereas it facilitates error detection and avoids interoperability problems such as byte-order (endianness), the processing time is increased in the serialize and deserialize steps and the logic to achieve the conversion can be cumbersome. Several options to overcome these problems are under discussion and several alternatives are emerging; attachments, MTOM [60] or external links are possible improvements for the performance when large data files are transmitted.

On the other hand, the stateless nature of the HTTP protocol may provoke time out responses when requests take too long to process, i.e: a typical grid use case of very intensive and lasting calculations. Asynchrony is necessary in those situations but the inherent statelessness of every request requires to implement some kind of polling mechanism to ensure that the results are retrieved back. An asynchronous response for DAS which also allows longer lasting computations and polling for results has been implemented within the Dasobert Java library [61]. A protocol based on HTTP cannot rely on it to manage state information, which in turn must be handled by the application layer, and until now DAS, REST or SOAP cannot be considered inherently asynchronous.

In contrast to the stateless RPC protocols that have been discussed, the XMPP protocol was initially considered for instant messaging applications.  But the usage of XMPP has been broadened to the whole realm of message oriented middleware. XMPP is an open standard promoted by many organizations and companies. The standard describes a flexible and extensible message passing mechanism which can be secured and encrypted. Other technologies, such as VoIP and file transfer signaling, have been based on XMPP. However, the protocol does have some perceived weaknesses: overheads associated to inefficient encoding and redundant transmission of presence data, as well as scalability issues that may emerge as the number of actors taking part in the communication increases. These could possibly harm the performance of the protocol, but in our opinion constitutes a relatively minor problem. Presence data are scarcely sent during the execution of distributed bioinformatics applications and instead users could be provided with real-time information about service availability. XMPP with IO Data (Wagener *et al*., submitted) is an interesting initiative that aims at delivering asynchronous, discoverable cloud services on top of XMPP, with services providing their own XML-Schema defining input output, and thereby eliminating the need for WSDL files.  Finally, a base64 encoding issue exists but could be overcome with any of two TCP/IP file transfer specifications over XMPP [62,63].  In general, sending large files still remains a cumbersome aspect of any communication protocol and a 'pass-by-reference' mechanism would be the most technically adequate.

In our future development plan for eBioX we have contemplated support for new commands of the DAS/1.53 specification as well as DAS/2. An effort towards the integration of different reference servers and their annotations with the help of ontologies or MD5 will be carried out. The user interface for the input of parameters, visualization features and memory management will be improved as well.

## Conclusion

We have reported the creation of an annotation server to distribute ERV data using the DAS protocol. Our preliminary tests prove the capabilities of the protocol and demonstrate the viability of combining reference and annotation servers. Our ERV data distribution can contribute to a better annotation of the retroviruses which remain widely unannotated or previously annotated as simple repeats. As with the TFRs areas, a rapid analysis of areas protected from transposon insertion was also facilitated by our server. Furthermore, considering that several human ERVs are interesting for their potential to cause alternative transcription or affect gene expression, distribution of ERV data using DAS could be used to gain insight into the retroviral adaptability. Finally, in order to understand the function and evolution of genomes, this server publicly releases an interesting dataset and makes ERV annotations easily integrable with genomic views at the same time. In that respect, we used eBioX, supporting a subset of the DAS/1.53E protocol, to provide visualization of ERV data as well as annotations from other sources. With this new DAS extension, eBioX gains the ability to participate in a service oriented architecture including annotation and reference providers.

## Availability and requirements

The source code of the DAS-ERV adaptors is available under the Lesser General Public License (LGPL) from . Requirements for installing these adaptors are a Perl interpreter and several modules including ProServer [38]. eBioX is available under the GPL license and can be downloaded from , where installation requirements are also stated.

## Competing interests

The authors declare that they have no competing interests.

## Authors' contributions

AMB implemented the DAS-ERV extensions to the DAS ProServer server and wrote the paper. EL is the main eBioX developer, implemented the code of the DAS extension with help of AMB and helped writing the paper. GS and JB, main designers and authors of RetroTector, provided the ERV data and substantial advice and guidance during all phases of the project. EBR planned and designed the project. All authors read and approved the final manuscript.

## Supplementary Material

Additional file 1 **Chromosomal coordinates of TFRs with overlapping retroviruses**. Tab delimited .bed file containing the hg18/NCBI 36 chromosomal coordinates (Chromosome, start, end, ID) for all human TFRs that overlap one of the annotated retroviruses. This data is also available in a browsable format at: . Click here for file

## References

[B1] Dowell RD, Jokerst RM, Day A, Eddy SR, Stein L (2001). The Distributed Annotation System. BMC Bioinformatics.

[B2] Lincoln D Distributed Annotation System (DAS) Version 1.53.

[B3] The BioSapiens project homepage. http://www.biodas.org.

[B4] ENFIN:: a European Network of Excellence for Data Integration and Systems Biology. http://www.enfin.org/page.php?page=home.

[B5] EMBRACE Network of Excellence. http://www.embracegrid.info/.

[B6] The Open-Bio DAS homepage. http://www.biodas.org.

[B7] Hubbard TJP, Aken BL, Beal K, Ballester B, Caccamo M, Chen Y, Clarke L, Coates G, Cunningham F, Cutts T, Down T, Dyer SC, Fitzgerald S, Fernandez-Banet J, Graf S, Haider S, Hammond M, Herrero J, Holland R, Howe K, Howe K, Johnson N, Kahari A, Keefe D, Kokocinski F, Kulesha E, Lawson D, Longden I, Melsopp C, Megy K, Meidl P, Ouverdin B, Parker A, Prlic A, Rice S, Rios D, Schuster M, Sealy I, Severin J, Slater G, Smedley D, Spudich G, Trevanion S, Vilella A, Vogel J, White S, Wood M, Cox T, Curwen V, Durbin R, Fernandez-Suarez XM, Flicek P, Kasprzyk A, Proctor G, Searle S, Smith J, Ureta-Vidal A, Birney E (2007). Ensembl 2007. Nucleic Acids Res.

[B8] Rogers A, Antoshechkin I, Bieri T, Blasiar D, Bastiani C, Canaran P, Chan J, Chen WJ, Davis P, Fernandes J, Fiedler TJ, Han M, Harris TW, Kishore R, Lee R, McKay S, Müller HM, Nakamura C, Ozersky P, Petcherski A, Schindelman G, Schwarz EM, Spooner W, Tuli MA, Van Auken K, Wang D, Wang X, Williams G, Yook K, Durbin R, Stein LD, Spieth J, Sternberg PW (2008). WormBase 2007. Nucleic Acids Res.

[B9] Stein LD, Mungall C, Shu S, Caudy M, Mangone M, Day A, Nickerson E, Stajich JE, Harris TW, Arva A, Lewis S (2002). The generic genome browser: a building block for a model organism system database. Genome Res.

[B10] GenoViz. Tools for Genomics Data Visualization. http://genoviz.sourceforge.net/.

[B11] OmniGene BioInformatics Page. http://omnigene.sourceforge.net/index.shtml.

[B12] Holland RCG, Down TA, Pocock M, Prlic A, Huen D, James K, Foisy S, Dräger A, Yates A, Heuer M, Schreiber MJ (2008). BioJava: an open-source framework for bioinformatics. Bioinformatics.

[B13] Stajich JE, Block D, Boulez K, Brenner SE, Chervitz SA, Dagdigian C, Fuellen G, Gilbert JG, Korf I, Lapp H, Lehväslaiho H, Matsalla C, Mungall CJ, Osborne BI, Pocock MR, Schattner P, Senger M, Stein LD, Stupka E, Wilkinson MD, Birney E (2002). The Bioperl toolkit: Perl modules for the life sciences. Genome Res.

[B14] Prlic A, Down TA, Kulesha E, Finn RD, Kahari A, Hubbard TPJ (2007). Integrating sequence and structural biology with DAS. BMC Bioinformatics.

[B15] DAS registration server – Distributed Annotation System. http://www.dasregistry.org/.

[B16] DAS version 1.53 Extended. http://www.dasregistry.org/spec_1.53E.jsp.

[B17] Jenkinson AM, Albrecht M, Birney E, Blankenburg H, Down T, Finn RD, Hermjakob H, Hubbard TJ, Jimenez RC, Jones P, Kähäri A, Kulesha E, Macías JR, Reeves GA, Prlic A (2008). Integrating biological data – the Distributed Annotation System. BMC Bioinformatics.

[B18] Help on coordinate systems. http://www.dasregistry.org/help_coordsys.jsp.

[B19] Jimenez RC, Quinn AF, Garcia A, Labarga A, O'Neill K, Martinez F, Salazar GA, Hermjakob H (2008). Dasty2, an Ajax protein DAS client. Bioinformatics.

[B20] Prlić A, Down TA, Hubbard TJ (2005). Adding some SPICE to DAS. Bioinformatics.

[B21] About Proview. http://www.efamily.org.uk/software/dasclients/proview/.

[B22] Ólason PI (2005). Integrating protein annotation resources through the Distributed Annotation System. Nucleic Acids Research.

[B23] Clamp M, Cuff J, Searle SM, Barton GJ (2004). The Jalview Java Alignment Editor. Bioinformatics.

[B24] PeppeR – Graphical 3D-EM DAS Client. http://biocomp.cnb.uam.es/das/PeppeR/.

[B25] DASMIweb – dynamic online integration and annotation of molecular interaction data. http://dasmi.bioinf.mpi-inf.mpg.de/.

[B26] DAS Ontology extension. http://www.dasregistry.org/extension_ontology.jsp.

[B27] Int Human Genome Sequencing Consort (2001). Initial sequencing and analysis of the human genome. Nature.

[B28] Bennetzen JL (2000). Transposable element contributions to plant gene and genome evolution. Plant Mol Biol.

[B29] Sandhu D, Gill KS (2002). Gene-containing regions of wheat and the other grass genomes. Plant Physiol.

[B30] Stoye JP (2001). Endogenous retroviruses: still active after all these years?. Curr Biol.

[B31] Katzourakis A, Pereira V, Tristem M (2007). Effects of recombination rate on human endogenous retrovirus fixation and persistence. J Virol.

[B32] Blikstad V, Benachenhou F, Sperber GO, Blomberg J (2008). Endogenous retroviruses: Evolution of human endogenous retroviral sequences: a conceptual account. Cell Mol Life Sci.

[B33] Han K, Sen SK, Wang J, Callinan PA, Lee J, Cordaux R, Liang P, Batzer MA (2005). Genomic rearrangements by LINE-1 insertion-mediated deletion in the human and chimpanzee lineages. Nucleic Acids Res.

[B34] Kazazian HH, Moran JV (1998). The impact of L1 retrotransposons on the human genome. Nat Genet.

[B35] Yu P, Zhang L, Li SF, Cheng JQ, Lu YR, Li YP, Bu H (2008). Transmission of porcine endogenous retrovirus to human cells in nude mouse. Acta Virol.

[B36] Xing J, Wang H, Belancio VP, Cordaux R, Deininger PL, Batzer MA (2006). Emergence of primate genes by retrotransposon-mediated sequence transduction. PNAS.

[B37] Deininger PL, Moran JV, Batzer MA, Kazazian HH (2003). Mobile elements and mammalian genome evolution. Curr Opin Genet Dev.

[B38] Jern P, Coffin JM (2008). Effects of Retroviruses on Host Genome Function. Annu Rev Genet.

[B39] Finn RD, Stalker JW, Jackson DK, Kulesha E, Clements J, Pettett R (2007). ProServer: a simple, extensible Perl DAS server. Bioinformatics.

[B40] ProServer DAS Server. http://www.sanger.ac.uk/Software/analysis/proserver/.

[B41] Distributed Annotation System. Retrieving the Stylesheet. http://www.biodas.org/documents/spec.html#stylesheet.

[B42] dasstyle.dtd. http://www.biodas.org/dtd/dasstyle.dtd.

[B43] Sperber GO, Airola T, Jern P, Blomberg J (2007). Automated recognition of retroviral sequences in genomic data – RetroTector. Nucleic Acids Res.

[B44] Custom annotation. http://www.ensembl.org/info/data/ensembl_das.html.

[B45] Simons C, Makunin IV, Pheasant M, Mattick JS (2007). Maintenance of transposon-free regions throughout vertebrate evolution. BMC Genomics.

[B46] Maintenance of transposon-free regions throughout vertebrate evolution. Supplementary data. http://jsm-group.imb.uq.edu.au/tfr07/.

[B47] Paces J, Pavlicek A, Zika R, Kapitonov VV, Jurka J, Paces V (2004). HERVd: the Human Endogenous RetroViruses Database: update. Nucleic Acids Res.

[B48] RetroSearch: ERV database (Annotated HERV ORFs in the human genome). http://www.daimi.au.dk/~biopv/herv/.

[B49] Villesen P, Aagaard L, Wiuf C, Pedersen FS (2004). Identification of endogenous retroviral reading frames in the human genome. Retrovirology.

[B50] RepeatMasker Home Page. http://www.repeatmasker.org/.

[B51] Kuhn RM, Karolchik D, Zweig AS, Wang T, Smith KE, Rosenbloom KR, Rhead B, Raney BJ, Pohl A, Pheasant M, Meyer L, Hsu F, Hinrichs AS, Harte RA, Giardine B, Fujita P, Diekhans M, Dreszer T, Clawson H, Barber GP, Haussler D, Kent WJ (2009). The UCSC Genome Browser Database: update 2009. Nucleic Acids Res.

[B52] Jurka J, Kapitonov VV, Pavlicek A, Klonowski P, Kohany O, Walichiewicz J (2005). Repbase Update, a database of eukaryotic repetitive elements. Cytogenet Genome Res.

[B53] Macías JR, Jiménez-Lozano N, Carazo JM (2007). Integrating electron microscopy information into existing Distributed Annotation Systems. J Struct Biol.

[B54] Jones P, Vinod N, Down T, Hackmann A, Kahari A, Kretschmann E, Quinn A, Wieser D, Hermjakob H, Apweiler R (2005). Dasty and UniProt DAS: a perfect pair for protein feature visualization. Bioinformatics.

[B55] Bose R, Mann R, Prina-Ricotti D (2006). AstroDAS: Sharing Assertions across Astronomy Catalogues through Distributed Annotation. Proceedings of the International Provenance and Annotation Workshop (IPAW'06).

[B56] DAS/2 – BioDAS. http://www.biodas.org/wiki/DAS/2.

[B57] RFC 3920 – Extensible Messaging and Presence Protocol (XMPP): Core. http://tools.ietf.org/html/rfc3920.

[B58] RFC 3921 – Extensible Messaging and Presence Protocol (XMPP): Instant Messaging and Presence. http://tools.ietf.org/html/rfc3921.

[B59] D3.1.1 Report on Technology Survey. http://www.embracegrid.info/files/pub/Deliverables/D3.1.1_v2.0.doc.

[B60] SOAP Message Transmission Optimization Mechanism. http://www.w3.org/TR/soap12-mtom/.

[B61] Dasobert DAS client library. http://www.spice-3d.org/dasobert/.

[B62] XEP-0096: SI File Transfer. http://xmpp.org/extensions/xep-0096.html.

[B63] XEP-0234: Jingle File Transfer. http://xmpp.org/extensions/xep-0234.html.

