# Supplementary material for: Annotation and visualization of endogenous retroviral sequences using the Distributed Annotation System (DAS) and eBioX
Source: BMC Bioinformatics. 2009 Jun 16;10(Suppl 6):S18. doi: 10.1186/1471-2105-10-S6-S18 (PMC2697641; doi:10.1186/1471-2105-10-S6-S18)
Supplement: Additional file 1 — Chromosomal coordinates of TFRs with overlapping retroviruses. Tab delimited .bed file containing the hg18/NCBI 36 chromosomal coordinates (Chromosome, start, end, ID) for all human TFRs that overlap one of the annotated retroviruses. This data is also available in a browsable format at: . [file 1471-2105-10-S6-S18-S1.pdf]

```
track name="TFRs" description="TFRs overlappande" visibility=2 height=10
1  151032935 151037988 hs1.427 449
2  241456628 241461844 hs2.839  622
4  3432254 3439213     hs4.46 649
4  65950622 65956070   hs4.171 319
4  191136189 191143152 hs4.562  568
8  7100402 7105460     hs8.36   554
8  7108025 7113082     hs8.37   552
8  7115647 7120704     hs8.38   553
8  7123269 7128326     hs8.39   555
8  7416312  7421373    hs8.46   552
8  7423961 7429017     hs8.47   552
8  93036041 93042555   hs8.303 390
10 87346722  87352869  hs10.225 474
11 5127533 5133755     hs11.65  354
X  140809758 140814770 hsX.275 499
```
